# One Big Happy Family? Unraveling the Relationship between Shared Perceptions of Team Psychological Contracts, Person-Team Fit and Team Performance

**DOI:** 10.3389/fpsyg.2017.01966

**Published:** 2017-11-09

**Authors:** Katherine Gibbard, Yannick Griep, Rein De Cooman, Genevieve Hoffart, Denis Onen, Hamidreza Zareipour

**Affiliations:** ^1^Department of Psychology, University of Guelph, Guelph, ON, Canada; ^2^Department of Psychology, University of Calgary, Calgary, AB, Canada; ^3^Division of Epidemiology, Stress Research Institute, Stockholm University, Stockholm, Sweden; ^4^Department of Work and Organisation Studies, University of Leuven, Leuven, Belgium; ^5^Schulich School of Engineering, University of Calgary, Calgary, AB, Canada

**Keywords:** team psychological contract breach, person-team fit, complementary fit, supplementary fit, team performance, time

## Abstract

With the knowledge that team work is not always associated with high(er) performance, we draw from the Multi-Level Theory of Psychological Contracts, Person-Environment Fit Theory, and Optimal Distinctiveness Theory to study shared perceptions of psychological contract (PC) breach in relation to shared perceptions of complementary and supplementary fit to explain why some teams perform better than other teams. We collected three repeated survey measures in a sample of 128 respondents across 46 teams. After having made sure that we met all statistical criteria, we aggregated our focal variables to the team-level and analyzed our data by means of a longitudinal three-wave autoregressive moderated-mediation model in which each relationship was one-time lag apart. We found that shared perceptions of PC breach were directly negatively related to team output and negatively related to perceived team member effectiveness through a decrease in shared perceptions of supplementary fit. However, we also demonstrated a beneficial process in that shared perceptions of PC breach were positively related to shared perceptions of complementary fit, which in turn were positively related to team output. Moreover, best team output appeared in teams that could combine high shared perceptions of complementary fit with modest to high shared perceptions of supplementary fit. Overall, our findings seem to indicate that in terms of team output there may be a bright side to perceptions of PC breach and that perceived person-team fit may play an important role in this process.

## Introduction

In today’s economic reality, colored by a range of organizational changes, downsizing, restructuring, lay offs, and rapid changing market needs, organizations need to constantly adapt in order to remain competitive and innovative. This evolution has given rise to greater reliance on teams to draw from the expertise of multiple individuals in an attempt to generate innovative solutions and produce results superior to what individual members would have accomplished independently (Fay et al., 2015; Salas et al., 2015). However, along the path to high performance, teams may encounter setbacks such as team conflict, that impinge upon their ultimate goal of producing high quality, innovative work (O’Neill et al., 2013; O’Neill et al., 2017). Although scholars are aware of the factors that may result in such setbacks, teams often still fail to reach their full potential (Salas et al., 2008). With the knowledge that team work is not always associated with high(er) performance, we draw from Multi-Level Theory of Psychological Contracts (Laulié and Tekleab’s, 2016), Person-Environment Fit Theory (Kristof-Brown and Guay, 2011), and Optimal Distinctiveness Theory (Brewer, 1991) to study teams’ shared perceptions of psychological contract breach in relation to shared perceptions of person-team fit to explain a team’s success or failure.

Psychological contracts (PC) are traditionally defined as “an individual’s beliefs regarding the terms and conditions of a reciprocal exchange agreement between that focal person and another party” (Rousseau, 1989, p. 123). PC breach occurs when individuals perceive that their employer does not meet one or more obligations at a specific point in time (Rousseau, 1989; Morrison and Robinson, 1997). Although the vast majority of PC research has centered on perceptions of PC breach at the individual level, a recent Multi-Level Theory of Psychological Contracts has proposed to study PCs at the team level to understand, among other things, how team PC fulfillment or breach relates to team performance (Laulié and Tekleab, 2016). Although this Multi-Level Theory of Psychological Contracts posits a direct negative relationship between team members collectively noticing that their team has failed to fulfill its obligations (team perceptions of PC breach) and team performance, we introduce person-team fit as an important mediator that will allow us to demonstrate why in certain incidences shared perceptions of PC breach will reduce team performance, while in other incidences it will actually increase team performance.

Person-team fit, which builds upon Person-Environment Fit Theory, proposes that it is not individual team members’ attributes that drive their functioning and well-being but the fit between these individual attributes and those of other team members. While person-team fit, has previously been conceptualized simply as congruence between the team and the individual (Kristof-Brown and Guay, 2011), recent work has stressed the differentiation between two types of person-team fit: complementary fit and supplementary fit (Edwards et al., 2006; De Cooman et al., 2016). De Cooman et al. (2016) have described *complementary fit* as an individual’s characteristics that form a unique addition to the team, while they refer to *supplementary fit* as an individual’s characteristics that are similar to the team. Based on recent insights in Person-Environment Fit Theory (e.g., DeRue and Morgeson, 2007; Edwards and Shipp, 2007; Kristof-Brown et al., 2014; Seong et al., 2015) stating that the subjective experience of different types of fit is influenced by work-related affective and behavioral events, we propose that in the event of PC breach, these fit types may be impacted differentially. We put forward that whereas shared perceptions of PC breach may decrease a collective sense of supplementary fit by revealing dissimilar expectations and contributions to the team (i.e., the traditional view in individual PC breach research), it may also increase a sense of complementary fit by creating a sensitivity for dissimilarities and unique contributions to the team. While the first proposition reflects a destructive process in terms of collaboration and team member likeability, the second proposition reflects a constructive process in terms of learning from each other, intellectual cross-pollination, and integrating and synergizing of diverse efforts. Next, based on Optimal Distinctiveness Theory (Brewer, 1991), which asserts that individuals are driven to reach an equilibrium between similarity and distinctiveness from their team, the sensitivity for unique dissimilarities that may add to the team (i.e., complementary fit) will be combined with a sensitivity for similarities and a striving for supplementary fit. Consequently, team members will believe that they bring unique additions to the team, while being similar enough to consider their team as one in which people are alike on important attributes. This combination of collectively perceived high levels of complementary and supplementary fit then increases team performance and as such may counter for the direct negative effect of shared PC breach and associated conflicts on team performance (Bell et al., 2011).

The contributions of this article are threefold. First, by integrating the literatures on PC and person-team fit and combining it with two different approaches on team performance (i.e., the objectively scored output and the subjectively peer-rated team effectiveness), we are able to develop and test a model that allows us to understand why some teams might be more successful than others. This is of critical importance because several scholars (e.g., Albrecht et al., 2015) have argued that individuals often operate in teams with the potential to create competitive advantages. Consequently, the mechanisms operating within the employee-employer relationship might be vastly different from the mechanisms operating at the team level, creating a need to potentially differentially manage team-level PCs. Moreover, traditional team effectiveness research only looks at either aggregated self-rated performance or objectively scored output. However, in order to fully capture the effectiveness of teamwork in a valid way it is crucial to take a broad view on performance by integrating team output or performance judged by relevant others external to the team, as well as the appreciation of peer-rated team effectiveness (i.e., linked to team viability; Hackman, 1987). Our study has thus the potential to inform policy makers and scholars about the importance of PCs at the team level as well as point them toward the important differences between complementary and supplementary fit perceptions in relation to internally and externally judged team performance.

Second, we introduce a temporal lens to the study of PC breach, person-team fit, and performance because recent theoretical and empirical developments have questioned the validity of PC and person-team fit as static concepts (e.g., Shipp and Jansen, 2011; Hansen and Griep, 2016). Moreover, a dynamic approach to the relationship between team perceptions of PC breach and team performance is warranted to investigate how key mediating mechanisms operate over time.

Finally, while there is an abundance of research on the negative effects of PC breach at the individual level (for a meta-analysis see Zhao et al., 2007), we know relatively little, if anything, about the role of PC breach at the team level. Our study contributes much-needed knowledge about team-level PC breach by being the first to empirically tests Laulié and Tekleab’s, (2016) Multi-Level Theory of Psychological Contracts. We will investigate the role of shared perceptions of PC breach in relation to team performance. By extending the literature of PC breach to a higher level of analysis, we are able to explain important organizational phenomena, such as team performance, beyond the traditionally studied individual level employee outcomes.

### Theoretical Framework and Hypotheses

#### Team Level Psychological Contracts

Although PC theory has traditionally been used to understand the mutual and reciprocal obligations in the employee–employer relationship, the seminal conceptualization of PC theory (Rousseau, 1989, 1995) already acknowledged that groups of individuals can develop a shared non-written agreement with their organization. Moreover, although PC theory (e.g., Rousseau, 1989, 1995, 2001) traditionally conceptualized the PC as an exchange relationship between an employee and his/her employer, it is important to note that from an underlying Social Exchange Theory (Blau, 1964) perspective both the employee and employer can be replaced by any two or more entities that engage in an exchange relationship with each other (e.g., team members among each other, therapist with patient, instructor with students). Fundamentally, a PC is an exchange agreement between multiple entities, with one such type of entity being the work team. To this effect, Marks (2001) moreover suggested that PCs in work teams may be more impactful than traditional employee-employer PCs because employees are increasingly dependent on their fellow team members to successfully complete a task than they are dependent on their organization as a whole.

When several individuals share a similar agreement and common beliefs about the content of their PC, Rousseau (1995) suggested that these individuals have a normative contract with their organization. However, ever since the initial proposition by Rousseau (1995), the idea of shared PCs has received little, if any, attention in the PC literature for a relatively long time. Similarly, the idea of the existence of a shared consensus on the extent to which a team develops shared perceptions of team PC breach has been understudied until recently. In the last decade, scholars have devoted substantial attention to the social context as an important factor that influences PC fulfillment or breach and the emergence of a team-level PC (e.g., Ho and Levesque, 2005; Ho et al., 2006; Dabos and Rousseau, 2013; De Vos and Tekleab, 2014; O’Leary-Kelly et al., 2014). In general, these scholars have suggested that shared perceptions of PC fulfillment and breach can be explained by social phenomena and continuous interactions among employees, such as team members.

These initial studies have facilitated the development of a new line of research focusing on PCs at the team level. Perhaps most influential in this sense is the work by De Vos and Tekleab (2014). Specifically, De Vos and Tekleab (2014) argued that social context influences the extent to which one perceives the PC as fulfilled or breached. As such, perceptions of PC fulfillment or breach can be shared among individuals who work closely together. They argued that the repeated interactions between team members facilitate the emergence of team perceptions of PC fulfillment or breach. Building on the initial work by De Vos and Tekleab (2014), Laulié and Tekleab’s, (2016). Multi-Level Theory of Psychological Contracts develops propositions on how perceptions of PC fulfillment and breach can be explained by social phenomena and continuous interactions among employees who work closely together (i.e., teams). Specifically, Laulié and Tekleab’s, (2016) introduce and differentiate two types of team-level PC constructs: *shared team PCs* and *shared individual PCs*. The former is defined as “the convergence of team members’ perception of the degree of fulfillment or breach of the obligations that an organization promised to the team” (Laulié and Tekleab, 2016, p. 662) whereas the later is defined as “the convergence of team members’ perception of the degree to which employers fulfill or breach their own individual psychological contracts” (Laulié and Tekleab, 2016, p. 663). Note that although the theoretical arguments for the emergence of both constructs are similar, they are conceptually and operationally different because the shared team PC deals with an aggregate of all team members’ perceptions of their team PC fulfillment or breach, whereas the shared individual PC deals with an aggregate of all team members’ perceptions of their own PC fulfillment or breach.

In the remainder of the paper, we will focus on *shared perceptions of team PC breach* as an aggregate of all team members’ perceptions of the extent to which their team breaches the team’s PC because we were interested in understanding how the obligations team members believed their team had toward each other were fulfilled or breached and how these shared team perceptions about PC breach influenced team performance. These shared team perceptions about PC breach may develop through multiple social interactions and information sharing among the team members (James and James, 1989). During these multiple interactions, team members might make promises to the team in return for some contributions by other team member. For example, team members might promise to provide a safe learning environment in which people are not mocked for mistakes or sufficient autonomy to each team member in return for team contributions such as timely delivery of products and services. As a result of these exchanges, team members might formulate expectations about each team member’s contributions to the team and the expected outcomes of these exchanges. Because perceptions of features, events, and processes tend to be shared among team members of a single team (e.g., Morgeson and Hofmann, 1999), team members of the same team are expected to develop shared perceptions of the degree to which promises and obligations made the overall team are fulfilled or breached.

#### Psychological Contracts and Performance at the Team Level

The Multi-Level Theory of Psychological Contracts (Laulié and Tekleab, 2016) proposes that the above mentioned shared perceptions of team PCs have the potential to directly influence a team’s performance. That is, several scholars (e.g., González-Romá et al., 2009; Bashshur et al., 2011) have demonstrated that team members, much in the same way as when individuals interact with their organization, desire to engage in positive social exchanges with each other.

In line with Social Exchange Theory (Blau, 1964) and the norm of reciprocity (Gouldner, 1960), team members engage in a mutual exchange relationship in which team members are expected to reciprocate the contributions of other team members by altering their own contributions either in a negative or positive way (Gouldner, 1960; Blau, 1964). As long as team members perceive that their team is fulfilling its obligations, the positive reciprocity norm (Gouldner, 1960) dictates that team members want to remain engaged in this social exchange relationship with their team and that they will reciprocate by enhancing their efforts to reach the team objectives, and increase their shared desire to perform effectively. By doing so, they may develop strong team goals which might make each team member more inclined to contribute to higher level endeavors, ultimately resulting in higher team performance (Blau, 1964; DeShon et al., 2004).

In contrast, when team members believe that their team failed to fulfill its obligations, these team members are more likely to reciprocate that behavior by reducing their personal contributions to the team because they feel exploited and outraged (Laulié and Tekleab, 2016). This argument aligns with Social Exchange Theory (Blau, 1964) and the negative reciprocity norm (Gouldner, 1960). That is, when a team member receives unfair treatment from its team (i.e., team members fail to fulfill their obligations), that team member is more likely to repay the team by, for example, reducing his/her performance to the team and endangering team output. Indeed, prior research, albeit at the level of the employee–employer relationship, has provided overwhelming empirical support for the argument that employees tend to reciprocate their organization’s failure to fulfill its obligations by reducing their performance (for a meta-analysis see Zhao et al., 2007). Extending this to PC breach at the team level, we argue that shared perceptions of team PC breach are likely to create a social climate that supports the withdrawal of effort and hence undermines team performance. Therefore, we hypothesize:

Hypothesis 1: Team members’ shared perceptions of psychological contract breach will be negatively related to peer-rated team effectiveness (H1a) and team output (H1b).

#### Introducing Person-Team Fit: Supplementary vs. Complementary Fit

Traditionally, the concept of person-team fit has hinged on similarity between an individual and their group and has been described as the perceived compatibility between individual team members and their team (Chatman, 1989; Kristof, 1996). Based on this definition, team members are expected to compare their psychological characteristics, such as values, goals, personality, needs and abilities, with their team members and construct a sense of fit within the team (Kristof-Brown A. et al., 2005). Although previous research argues that person-team fit emerges solely based on likeness and therefore high homogeneity within a team is ideal for team performance (Kristof-Brown and Guay, 2011), research shows that specific assessments of fit tend to be highly inter-correlated (Kristof-Brown A.L. et al., 2005) and that a superordinate person-team fit construct drives the more specific person-team fit assessments (Seong and Kristof-Brown, 2012). This implies that perceived person-team fit can be derived from either perceived similarity, perceived complementary, or even both (Muchinsky and Monahan, 1987). Building on this work, Piasentin and Chapman (2007) and De Cooman et al. (2016) introduced a more nuanced understanding of person-team fit that advances two types of person team fit: complementary and supplementary person-team fit.

From a *supplementary person-team fit* perspective, fit occurs when there is high similarity between a team member’s psychological characteristics and the other team members. The underlying theoretical idea is based on the similarity-attraction paradigm (Byrne, 1971) which states that a team member is more likely to be attracted to, and like, team members who are more similar to themselves because these relationships are believed to be more rewarding and supportive (Byrne, 1971; Cable and Edwards, 2004). Several empirical studies indeed support this idea by demonstrating a positive relationship between perceived supplementary fit and co-worker satisfaction, team cohesion, general satisfaction, organizational commitment, organizational citizenship behaviors and a negative relationship with turnover intensions (e.g., Kristof-Brown A.L. et al., 2005; Guan et al., 2011). In contrast, from a *complementary person-team fit* perspective, fit occurs when a team member possesses psychological characteristics that are unique and unlike the characteristics of the other team members. This team member perceives that (s)he differs from the other team members on important criteria and by doing so, this dissimilarity makes him/her unique and a valued member of the team (Piasentin and Chapman, 2007). The underlying theoretical idea is based on the psychological process of need fulfillment (Edwards, 1991) which states that a weakness of a team can be compensated for by the strength of other team members in the work environment and vice versa. Indeed, empirical research has demonstrated that perceived complementary fit relates positively to organizational citizenship behaviors, organizational commitment, and relates negatively to turnover intentions (Piasentin and Chapman, 2007; Guan et al., 2011).

Based on recent insights in Person-Environment Fit Theory (DeRue and Morgeson, 2007; Edwards and Shipp, 2007; Kristof-Brown et al., 2014; Seong et al., 2015) fit perceptions (i.e., the subjective experience of person-environment fit) do not only impact upon attitudes and behaviors but are also formed based on work-related affective and behavioral events such as experiences with performance feedback and momentary job satisfaction. Shared perceptions of PC breach are arguable one of the most impactful work-related affective events that may influence momentary experiences of supplementary and complementary fit. Besides this general idea of reversed causality and interest in antecedents of perceived fit a push toward a group or team-level approach to person-team fit (e.g., DeRue and Hollenbeck, 2007; Edwards and Shipp, 2007; Kristof-Brown et al., 2014; Seong et al., 2015) is noticed. Here, authors argue that an environment like a team is a complex system, which implies that shared perceptions of person-team fit may play a crucial role when explaining team effectiveness. Recent studies (e.g., Kristof-Brown et al., 2014; Seong et al., 2015) indeed support the relevance of perceived person-team fit as a meaningful group-level concept by showing that team-interactions produce a collective fit experience that influences individual and unit-level outcomes. In other words, while individual team members may perform well or feel good when they believe that they fit to their team, the team as whole will only perform well when all team-members share the same perceptions of team fit; hence the importance of shared perceptions of team-fit.

#### Shared Team-Level Psychological Contracts and Person-Team Fit

Traditionally, scholars have approach perceptions of PC breach as an adverse event that decreases favorable attitudes and behaviors among employees. In line with this, we propose that shared perceptions of PC breach create a sense of displeasure due to violations of the collectively formed expectations within the team which then may lead to a decrease in members’ perceptions of supplementary fit and finally result in lower appreciation of the qualities and efforts of different members of the team. Shared perceptions of PC breach may thus drive individuals to perceive themselves as more distinct from their team because they may not want to perceive themselves as closely aligned with a team where negative events are the norm. This is a negative process in which a team reflects on the process by thinking “Well, we thought that we would be able to function as a well-oiled team, but apparently we do not match the team as well as we thought.” Indeed, past research has found that when PC breaches occur, employees experience decreased person-organization fit (Bocchino et al., 2003). Bocchino et al. (2003) operationalized fit as congruence between an employee’s and an organization’s values, which is analogous to supplementary person-team fit. Besides the negative effect on shared perceptions of supplementary fit, shared perceptions of PC breach may impact perceptions of complementary fit in a different way. Shared perceptions of PC breach may, over time, create a sense of complementary within the team because team member get focused on differences and how these differences may contribute to the final goal of the team, i.e., the team effectiveness. This is a positive process in which a team reflects on the process by thinking “Well, things do not go as planned, but we are stuck in this team and collectively responsible for the output. Hence we focus on what each team member may serve to the team to ensure a timely and qualitative output.” In combination, these arguments lead us to the following hypothesis:

Hypothesis 2: Team members’ shared perceptions of psychological contract breach will be negatively related to team members’ shared perceptions of supplementary person-team fit (H2a), and team members’ shared perceptions of psychological contract breach will be positively related to team members’ shared perceptions of complementary person-team fit (H2b).

#### Person-Team Fit and Team Performance

The impact of perceived supplementary fit is theoretically based on the similarity-attraction paradigm (Byrne, 1971) in which a person is attracted to, and more inclined to like, others similar to themselves because these relationships are more rewarding and supportive (Cable and Edwards, 2004). Indeed, empirical evidence supports the relationship between perceived supplementary person-fit and co-worker focused outcomes (e.g., co-worker satisfaction, cohesion), work attitudes (e.g., satisfaction, organizational commitment, and turnover intentions), and behaviors (e.g., OCB) (Kristof-Brown A.L. et al., 2005; Guan et al., 2011). A decrease in shared perceptions of supplementary fit may thus negatively influence peer-rated team effectiveness. Moreover, scholars have argued that teams who are more heterogeneous, and thus characterized by higher complementary fit, tend to outperform their more homogeneous counterparts (Nemeth, 1986). Early work by Hoffman and Maier (1959) found that for complex problems, teams with more diverse personalities capitalized on having a broader range of perspectives and ideas, and this resulted in heterogeneous groups generating higher-quality solutions than homogenous ones. Additionally, teams with a wider base of functional diversity have been found to perform better (Bell et al., 2011). When a team is faced with a complex, multifaceted problem, having a diverse pool of knowledge to draw upon (i.e., high perceptions of complementary person-team fit) may be beneficial for performance. The premise by which heterogeneous teams outperform heterogeneous teams is that divergent thinking will create productive conflict. These findings have been supported for various types of group heterogeneity, including expertise (Stasser et al., 1995), and information (Gruenfeld et al., 1996). With respect to person-team fit this argument also received empirical support by Kristof-Brown A.L. et al. (2005), concluding that personality complementarity may induce individuals to contribute more fully to team-based work. Taken together, these findings support the notion that teams who perceive themselves as more complementary will see performance advantages due to their varied backgrounds and the productive conflict that may ensue as a result. Therefore, we hypothesize:

Hypothesis 3: Team members’ shared perceptions of supplementary person-team fit will be positively related to peer-rated team effectiveness (H3a), and team members’ shared perceptions of complementary person-team fit will be positively related to team output (H3b).

Yet, Brewer’ (1991) Optimal Distinctiveness Theory posits that members of a group aim to reach an equilibrium between the amount that they feel similar to and distinct from their group. Building on this hypothesis, Piasentin and Chapman (2007) and De Cooman et al. (2016) argued that at any given moment, a team member might perceive supplementary *and* complementary fit to his/her team, hence warranting the examination of an interaction between both types of person-team fit (Ostroff, 2012). Brewer’ (1991) Optimal Distinctiveness Theory sheds light on how these two types of person-team fit may coexist and interact. This theory posits that people have a desire to belong and be immersed in a social group (strive for high levels of supplementary person-team fit) and at the same time have a desire to distinguish themselves from other persons in a social context (strive for high levels of complementary person-team fit). According to Optimal Distinctiveness Theory (Brewer, 1991), when only one of these needs—either high supplementary person-team fit or high complementary person-team fit—is satisfied, team members will perceive that their sense of security, self-worth, and identity are threatened. Therefore, Optimal Distinctiveness Theory (Brewer, 1991) posits that team members will seek to achieve an equilibrium between feeling similar to their group and distinct from their group in order to build a strong team identity that will contribute to positive attitudes and behaviors toward the team. Based on these theoretical arguments, we assume an interaction between perceptions of supplementary and complementary person-team fit in such a way that perceptions of supplementary person-team fit have the ability to boost the positive relationship between perceptions of complementary person-team fit and team performance. Therefore, we hypothesize:

*Hypothesis 4: Perceptions of peer-rated member effectiveness (H4a) and team output (H4b) will be highest when team members have high shared perceptions of both complementary and supplementary person-team fit*.

## Materials and Methods

### Procedure and Participants

This study was approved by the Conjoint Faculties Research Ethics Board (REB16-1932) of the second author’s institute. We contacted 217 final year engineering students at a major Canadian University, of which 158 students (72.81% response rate) completed the first wave of data collection, 127 students completed the first and second wave of data collection (58.52% response rate), and 123 students (56.68% response rate) completed all three waves of data collection; ultimately resulting in 369 observations. We conducted logistic regression analyses to estimate differences between our final sample and dropouts and found that none of the demographics nor the variables under study explained dropout during different measurement occasions; implying that dropout occurred randomly.

At the start of the semester, students were divided into project teams with four to eight members who collaborated intensively, meeting at least once per week, on a collective project for a period of several weeks. During their first meeting, they were instructed to talk about their expectations they held toward their team, by doing so they were developing a PC at the team level. Every meeting thereafter, they worked on their collective project. The project involved solving a real-world engineering problem and creating a prototype. Students were required to follow a rigorous engineering design process, which involved developing a concept, engaging in project management, testing their prototype, writing technical documentation, and presenting the project. The project work was responsible for nearly the entirety of their final grade. We collected 3-wave data from these students independent from the course instructor (i.e., the course instructor was not given access to the survey data). In addition, we asked the course instructor to provide us with a list of the administrated collective team grades (third-party measure of team output) at the end of the semester.

### Measures

*Perceived PC breach* was measured using three statements that directly measure PC breach (see Vantilborgh et al., 2016). Respondents were presented with the following statements: (1) *The team has done a good job of meeting its obligations to me*; (2) *The team has repeatedly failed to meet its obligations to me*; and (3) *The team has fulfilled the most important obligations to me*. We asked our respondents to rate these items on a 7-point Likert scale ranging from (1) “Strongly disagree” to (7) “Strongly agree.” Items 1 and 3 were reverse-coded. We used this global measure of PC breach because several scholars found no significant difference in reactions to perceptions of PC breach when measured with a facet-based measure or with a global measure of PC breach, leading the authors to conclude that global measures of PC breach are preferable to facet level measures of PC breach when using a repeated measurement survey design as the one used in the current study (Bordia et al., 2008; Griep et al., 2016; Vantilborgh et al., 2016). An additional benefit to the use of such a concise global measure of PC breach over a longer facet-based measure pertains to the fact that respondents are less likely to drop out when having to complete this survey multiple times over the course of a short time span.

*Perceived person-team fit* was measured using Piasentin and Chapman’s (2007) multidimensional measure of perceived fit. This measure consists of 17 items: nine items to assess supplementary person-team fit and eight items to assess complementary person-team fit. We slightly adjusted the wording of the items to better capture the person-team fit instead of the person-organization fit. Specifically, we changed the words “employees or coworkers” into “team members” and changed the words “organization or company” into “team.” We asked our respondents to rate all items on a 7-point Likert scale ranging from (1) “Strongly disagree” to (7) “Strongly agree.” The level-specific within-person (ω = 0.92 and ω = 0.90, respectively) and between-person (ω = 0.91 and ω = 0.89, respectively) omega reliability (Geldhof et al., 2014) was satisfactory.

*Team output* was operationalized as the grade that teams received for their project work. These grades were assigned by the course instructor and could range from 0 (very low performance) to 100 (very high performance). In addition, we collected *peer-rated team effectiveness ratings*, which were peer-rated evaluations of all team member’s abilities on five competencies that have been found to be of critical importance for team effectiveness. The five competencies are: (1) Commitment to the team’s work; (2) Communicating with team members; (3) Having a strong foundation of knowledge, skills and abilities; (4) Emphasizing high standards; and (5) Keeping the team on track (Loughry et al., 2007). In the survey, each of the five competencies included detailed descriptions and students were asked to rate each competency for each of their fellow teammates. We asked our participants to rate all items on a 5-point Likert scale ranging from (1) “To no extent” to (5) “To a great extent.” For the purpose of this study, we calculated a *general peer-rated team effectiveness rating* by averaging across the five competences. The level-specific within-person (ω = 0.98) and between-person (ω = 0.91) omega reliability (Geldhof et al., 2014) was satisfactory.

### Analysis

Although most research to date has approached perceptions of PC breach and team-fit as individual level phenomena, there is a growing body of literature pointing toward the validity of a team-level approach to these phenomena (for an elaborate discussion see our literature review). In line with this novel conceptualization of PC breach and team-fit perceptions as a group construct, we adhere to the analysis criteria outlined by the Multi-Level Theory of Psychological Contracts (Laulié and Tekleab, 2016) to align our theoretical framework with our analytical approach. Specifically, we assessed the between-team variance using one-way analysis of variance and the intraclass coefficient ICC1 to establish whether investigation of team-level relationships was warranted (Hofmann et al., 2000). As can be seen in **Table 1**, all F-coefficients were significant, and the ICC1 values were medium in size (LeBreton and Senter, 2008). To further establish team-level properties of our focal variables, we estimated the median *r*_wg_ to assess homogeneity of our focal variables within different teams (James et al., 1984). As can be seen in **Table 1**, we found adequate levels of agreement (*r*_wg_ ranging from 0.73 to 0.96); providing further support for the team-level structure of our focal variables. Finally, we assessed the ICC2 values of all focal variables, and found them to be adequate (>0.70). In combination, this warrants the exploration of team sources of variance and allow for the aggregation of our focal variables to the team-level so that we can investigate our hypothesized relationships at the team-level.

**Table 1:** Means, standard deviations, and correlations at the team level.

	*M*	*SD*	*F*	ICC1/ICC2	*R*_wg_	1	2	3	4	5
(1) Psychological contract breach T1	1.65	0.44	64.13^∗∗∗^	0.16/0.84	0.86	–				
(2) Supplementary fit T2	5.42	0.72	69.93^∗∗∗^	0.15/0.89	0.76	0.10	–			
(3) Complementary fit T2	4.89	0.63	81.56^∗∗∗^	0.12/0.84	0.73	0.43^∗∗^	0.52^∗∗∗^	–		
(4) Peer-rated team effectiveness T3	4.49	0.36	29.92^∗∗∗^	0.07/0.85	0.96	0.07	0.21	0.39^∗∗^	–	
(5) Team output T3	91.08	5.57	24.62^∗∗∗^	0.27/0.96	0.74	–0.11	0.05	–0.07	0.05	–

*N_*teams*_ = 48; *N*_*individuals*_ = 123. ^∗^*p* < 0.05, ^∗∗^*p* < 0.01, ^∗∗∗^*p* < 0.001.*

After having aggregated our focal variables to the team-level, we analyzed our data by means of a longitudinal three-wave autoregressive moderated-mediation model (Cole and Maxwell, 2003; MacKinnon, 2008) in which each relationship is one time lag apart (i.e., relationships from Time 1 to Time 2, and from Time 2 to Time 3). We followed the recommendations of Edwards and Lambert (2007) and simultaneously tested the moderation and mediation effects. The moderation effects were tested by including an interaction effect between shared perceptions of supplementary and complementary fit at Time 2. To facilitate the interpretation of this moderation, we grand-mean centered the moderator and relied on the simple slopes method (i.e., interaction effects for -1SD, mean, +1SD of the moderator). We tested the mediation effects by means of the product-of-coefficients approach (i.e., the product of each a-path with each b-path). By investigating these longitudinal relations between shared perceptions of team PC breach at Time 1 and shared perceptions of supplementary and complementary fit at Time 2 and team performance at Time 3, we investigate the temporal precedence of the mediation effect. We drew 10,000 bootstrap samples to generate 95% bias-corrected confidence intervals (95% CIbc; Preacher et al., 2007) around the indirect effects. All analyses were conducted in Mplus version 7.1 (Muthén and Muthén, 2013) with team size, individual variance in perceptions of supplementary and complementary fit within a team, variance in peer-rated team effectiveness within a team, and variance in perceptions of PC breach within a team as control variables.

## Results

### Descriptive Results

**Table 1** presents means, standard deviations, and correlations of the study variables at the aggregated team level.

### Model Comparison

Prior to presenting the results, we assessed whether a full or partial mediation model fits the data best. Based on the BIC and sample-size adjusted BIC value, the full moderated-mediation model fits the data best (BIC = 811.35; sample-size adjusted BIC = 695.34) compared to the partial moderated-mediation model (BIC = 818.84; sample-size adjusted BIC = 696.55). Hence, the full moderated-mediation model will guide our hypotheses testing^1^.

### Hypothesis Testing

**FIGURE 1:**
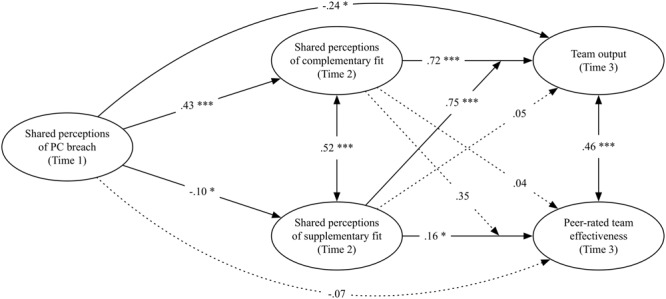
Standardized estimated paths in the longitudinal three-wave autoregressive full moderated-mediation model. ^∗^*p* < 0.05, ^∗∗^*p* < 0.01, ^∗∗∗^*p* < 0.001. Dotted lines indicate non-significant relationships. Double arrowed lines indicate correlations. Results indicate change in each variable by controlling for the auto-correlation at the previous moment in time. The relationships from shared perceptions of PC breach to team output and peer-rated team effectiveness are part of the partial moderated-mediation model and are presented as per reviewer’s request.

**Figure 1** displays the results of the longitudinal three-wave autoregressive full moderated-mediation model with team size, individual variance in perceptions of supplementary and complementary fit within a team, variance in peer-rated team effectiveness within a team, and variance in perceptions of PC breach within a team as control variables. Because our the full moderated-mediation model fits the data better, we are unable to test the direct effect from team members’ shared perceptions of PC breach at Time 1 to team performance or peer feedback ratings at Time 3 (Hypothesis 1). However, as per reviewer suggestions, we did inspect this direct effect in the partial moderated-mediation model and found a negative direct effect of team members’ shared perceptions of PC breach at Time 1 on team output [*B* = -0.235, 95% CI = (-0.444, -0.027)] at Time3, but no significant relationship with peer-rated team effectiveness [*B* = -0.074, 95% CI = (-0.218, 0.366)] at Time 3. These results are in line with Hypothesis 1b, but are not in line with Hypothesis 1a.

Moreover, our results indicated that team members’ shared perceptions of team PC breach at Time 1 were negatively related to team members’ shared perceptions of supplementary fit at Time 2 [*B* = -0.102, 95% CI = (-0.441, -0.237)], whereas team members’ shared perceptions of team PC breach at Time 1 were positively related to team members’ shared perceptions of complementary fit at Time 2 [*B* = 0.432, 95% CI = (0.210, 0.653)]. These results are in line with Hypothesis 2a and 2b.

Furthermore, our results indicated that team members’ shared perceptions of complementary fit at Time 2 were positively related to team output at Time 3 [*B* = 0.368, 95% CI = (0.129, 0.606)] and were not significantly related to peer-rated team effectiveness ratings at Time 3 [*B* = 0.148, 95% CI = (-0.127, 0.422)]. In contrast, we found that team members’ shared perceptions of supplementary fit at Time 2 were positively related to peer-rated team effectiveness ratings at Time 3 [*B* = 0.163, 95% CI = (0.098, 0.228)], and were unrelated to team output at Time 3 [*B* = 0.053, 95% CI = (-0.182, 0.288)]. In addition, we found a positive time-lagged indirect effect (i.e., predictor during Time 1 to mediator at Time 2, and mediator at Time 2 to outcomes at Time 3) of team members’ shared perceptions of team PC breach to team output via c [*B* = 0.132, 95% CI = (0.015, 0.250)]. All other time-lagged indirect effect were not significant. These results support Hypothesis 3a and 3b.

Finally, we found a positive conditional indirect effect of team members’ shared perceptions of team PC breach on team output via team members’ shared perceptions of complementary fit when team members’ shared perceptions of supplementary fit were both low [*B* = 0.734, 95% CI = (0.105, 1.363)], and high [*B* = 1.619, 95% CI = (0.093, 3.145)], implying that the mediation effect grew stronger as team members’ perceptions of supplementary fit increased. These results partially support Hypothesis 4b but do not support Hypothesis 4a.

## Discussion

In this study, we set out to unravel the longitudinal relationship between team members’ shared perceptions of team PC breach, person-team fit, and team performance to better understand why some teams achieve high performance while others do not. Specifically, we drew from the Multi-Level Theory of Psychological Contracts, Person-Environment Fit Theory and Optimal Distinctiveness Theory to understand the impact of shared perceptions of PC breach on both internally rated and externally rated team performance outcomes and the potential mediating roles of complementary and supplementary fit. By extending the literature of PC breach and person-team fit to a higher level of analysis, we are able to explain important organizational phenomena, such as team performance, beyond the traditionally studied individual level outcomes. In doing so, our study presents three major contributions to the literature on PCs and person-team fit.

First, although PC research has primarily been limited to the individual level of analysis, we demonstrated, in line with Laulié and Tekleab’s Multi-Level Theory of Psychological Contracts (2016), that perceptions of PC breach tend to be influenced by the interactions between team members. Our findings indicate that, contrary to the traditional social exchange viewpoint in which PC breach is a unilateral exchange, the dynamics within teams alter how PC breaches are perceived. That is, interactions between team members seem to shape the interpretation of team PC breach and potentially have a differential influence (i.e., PC breach does not always reduce team performance; it depends on shared perceptions of person-team fit and on the type of performance outcome) on outcomes compared to the individual level (i.e., PC breach reduces individual performance). It thus seems that team members jointly shape the interpretation of, and reaction to, PC breach at the team level. By doing so the current manuscript contributes to the scant research on perceptions of PCs at the team level by providing a clear understanding of the direct and indirect effects of shared perceptions of PC breach and performance at the team level. This does not only adhere to Laulié and Tekleab’s, (2016) argument that researchers need to understand the role of shared team perceptions of PCs in relation to team outcomes, but it also contributes to a growing body of literature demonstrating how social exchange indicators operate at higher levels of analysis (i.e., teams). Specifically, it seems that, akin to operationalizing the PC as an individual’s mental model (Rousseau, 2001), the PC operates as a team’s mental model or “team members’ shared, organized understanding and mental representation of knowledge about key elements of the team’s relevant environment” (Mohammed et al., 2010, p. 879). Much like individual mental models, these team mental models of the PC could be used to better understand a team’s functioning.

Second, although the person-team fit literature has predominantly focused on the individual perspective and on supplementary person-team fit perspective (for some exceptions see Kristof-Brown A.L. et al., 2005; Seong and Kristof-Brown, 2012; Seong and Choi, 2014), our study answers Kozlowski et al.’s (2013) and Seong and Choi’s (2014) call for more research on the emerging nature of group-level fit perceptions by demonstrating that team member’s shared perceptions of PC breach trigger an increase in team members’ shared perceptions of complementary fit, while simultaneously triggering a decrease in team members’ shared perceptions of supplementary fit. Our findings confirm that, although complementary fit is less considered in person-environment studies, it is an important variable to understand the effect of negative experiences in the workplace as holding the potential of being positively related to team performance. The positive association between these increased shared perceptions of complementary fit and team performance (i.e., team output) aligns with past work that has demonstrated the superior performance experienced by heterogeneous teams as compared to homogeneous teams. These studies argue that as teams perceived themselves to be more diverse, the constructive conflict that results from their more varied ideas and backgrounds can result in performance gains (e.g., Nemeth, 1986; Stasser et al., 1995; Bell et al., 2011). Related to these proposed performance benefits of heterogeneous teams, we hypothesized that lower supplementary fit would be associated with lower peer-rated team effectiveness. This finding aligns with past research demonstrating that supplementary fit is important for positive perceptions of one’s team or social group and their subjective performance perceptions (Kristof-Brown A.L. et al., 2005), even if their actual performance would not necessarily bear out similar results. This finding emphasizes the relevance of disentangling team output and perceived team effectiveness as two different criteria of team effectiveness and performance (as proposed by Hackman, 1987); the former being crucial for goal attainment of the current team and the later being crucial for future team work by influencing the willingness to participate and contribute to future team work based on the experienced effectiveness of the current team. These results demonstrate the added value of considering complementary fit in addition to the more frequently measured supplementary fit when trying to understand team effectiveness. Our results demonstrated that the relationships between shared perceptions of PC breach and team performance depend upon the form of performance that is considered (i.e., internally or externally rated performance). That is, when we would only focus on self-rated performance (peer-ratings of team effectiveness), we would conclude that team member’s shared perceptions of PC breach endangers team effectiveness. However, when we focus on objective team performance (i.e., team output), we can conclude that team member’s shared perceptions of PC breach have the potential to boost team performance through increased shared perceptions of complementary fit and through the interaction of high shared perceptions of complementary fit and modest to high shared perceptions of supplementary fit.

Third, we demonstrated that the interaction between shared perceptions of complementary and supplementary fit matters in predicting team performance. Specifically, we found that team members’ shared perceptions of supplementary fit positively moderated the positive relationship between team members’ perceptions of complementary fit and team output. This finding is in line with Optimal Distinctiveness Theory (Brewer, 1991) proposition that team members will seek to achieve an equilibrium between feeling similar and distinct from their group. Hence, based on this results we can conclude that, in order to achieve high team output, it is important for team members to believe that they bring unique additions to the team, while having a baseline of similarity to consider the team as good fitting team in which people are alike on important attributes.

### Limitations

Like all studies, our study has limitations that deserve further attention. First, our data were collected using three repeated measurement surveys in which we collected all variables at the same point in time (although presented in a random order in each survey). Although this might raise concerns with common method variance (Podsakoff et al., 2012), we estimated a time-lagged model in which our predictor, mediators and outcomes were one time-lag apart and used both peer-rated and other-rated performance data to reduce the risks owing to common method bias and common rather effects. In addition, Siemsen et al. (2010) argued that common method bias cannot explain interactions effects. The presence of significant interactions in our study thus helps to strengthen our argument that the observed relations are a function of the studied constructs and relationships rather than methodological artifacts.

A second limitation concerns the self-report nature of the data for perceptions of team PC breach, complementary and supplementary fit, and peer ratings of team effectiveness. Although it has often been suggested to rely on other-rated measures to overcome the issue of social desirability, the concepts under study are idiosyncratic in nature and thus often unobservable by others. Therefore, we relied on self-reported measurements, but aimed to minimize social desirable responses by allowing discretionary participation and by guaranteeing the confidentiality (see Berry et al., 2012). However, we also collected third-party ratings of performance to further triangulate our data (i.e., peer- and other-reported data).

A third limitation concerns our sample size and use of students. Although our sample size at the individual (*N* = 123) and team level (*N* = 48) was relatively small, it should be noted that we had 369 observations and adequate power to detect medium and large effects at our level of analysis (i.e., team level). Related to our sample, we would also like to be cautious when generalizing our findings to employee teams because our student teams were all relatively young and had little previous work experience. We thus recommend future studies to study employee teams in an organizational setting in an attempt to replicate these findings.

A final limitation concerns the use of only three time points to study the proposed relationships. Although we analyzed the data using a longitudinal three-wave autoregressive moderated-mediation model, allowing us to model change from one wave to the next wave, we are unable to demonstrate how growth patterns in one variable relate to growth patterns of another variable over time. To answer these research questions, we advice future research to use experience sampling designs, in which respondents are surveyed at random intervals throughout the day (Fisher and To, 2012).

### Suggestions for Future Research

Although our study provides initial evidence for the important role of team members’ shared perceptions of complementary and supplementary fit in the relationship between team members’ shared perceptions of PC breach and team performance, future research is needed to validate this novel team-level approach. Most importantly, if shared perceptions of PC breach at the team level are not necessarily detrimental for a team’s performance (i.e., team output) because it increases a team’s level of complementary fit, a natural next step would be to examine the circumstances under which these shared perceptions of team PC breach have the potential to be beneficial for a team’s performance. We propose that such a variable of interest may be psychological safety. Specifically, we propose that in teams with high levels of psychological safety, team members will experience increased instances of interpersonally risky learning behavior, such as help seeking and discussion of errors (Edmondson, 2002). In instances of team PC breach, individuals who feel comfortable sharing their honest opinions within the team may be more likely to admit that a PC breach had occurred, and may feel more comfortable acknowledging that they form a unique addition to the team (i.e., increased complementary fit) instead of being similar to the team. Hence, members of teams characterized by high scores on psychological safety will more honestly appraise a negative team experience like team PC breach and perceive this team PC breach as less negatively impactful. There is indeed some empirical support for the association between psychological safety and reporting negative events. For example, Edmondson (1999) found that high performing teams reported higher error rates than lower performing teams. However, this wasn’t due to the higher performing teams making more errors – it was due to the higher performing teams feeling safe to admit to their errors. It thus seems that teams with higher levels of psychological safety more openly admit their failings, which may enable them to more honestly disclose occurrences of team PC breach and in turn more effectively deal with this negative occurrence.

### Practical Implications

These findings have important implications for teams as they strive for high performance. The finding that team members’ shared perceptions of team PC breach triggers increased perceptions of complementary fit, and in turn better performance (i.e., higher grades), indicates that there may in fact be a bright side to shared perceptions of PC breach at the team level. While individual level PC breach may lead to negative emotional, attitudinal, and behavioral consequences (Zhao et al., 2007), our findings indicate that having shared perceptions of PC breach at the team level may operate differently and have the potential to lead to positive outcomes via increased shared perceptions of complementary fit. Team members’ perceptions of complementary fit allows an event that initially appears negative (i.e., PC breach) to result in tangible positive outcomes for the team due to the team’s increased level of heterogeneity and diversity (Nemeth, 1986; Bell et al., 2011). It thus seems that the often heard advice to “prevent perceptions of PC breach and its ensuing violation feelings from occurring” may not always apply to teams. However, we do need to point out that these shared perceptions of complementary fit at the team level were also found to decrease peer-rated team effectiveness. This is an important caveat for teams with greater perceived diversity because it indicates that while objective ratings of performance, such as team output, may benefit from increased perceptions of complementary fit in the aftermath of PC breach, subjective perceptions of team well-being may suffer. In other words, while the team may objectively perform better due to increased shared perceptions of complementary fit, their experience within the team may suffer.

Tying in these two perspectives on team performance (i.e., objective performance and subjective team well-being), it seems that organizations need to strive for an optimal balance between complementary and supplementary fit. We believe that this can be achieved by building a psychological safe environment (see suggestions for future research) as well as by reinforcing complementary and supplementary fit when creating teams. Specifically, this implies that organizations need to pay attention to the extent to which employees resemble and complement other team members with regards to certain characteristics (e.g., personality traits, competences, skills). Our results indeed indicated that shared perceptions of complementary (i.e., higher team output) and supplementary (i.e., higher peer-rated team effectiveness) fit are relevant for team outcomes, and that a baseline level of supplementary fit is required to achieve high performance. Hence, we would advise organizations to provide ample opportunities for social interaction through for example onboarding and team-building activities (Klein and Kozlowski, 2000; Klein et al., 2009) because these actions have been shown to increase the emergence of shared perceptions of both types of person-team fit.

## Conclusion

Our findings largely provide support for the proposed relationships between team member’s shared perceptions of PC breach and team performance as influenced by team member’s shared perceptions of complementary and supplementary fit. Specifically, our findings demonstrate that shared perceptions of team PC breach are not necessarily a bad thing for team performance because shared perceptions of team PC breach seem to benefit team members’ shared perceptions of complementary fit, which was positively related to team output. However, we also found that a certain baseline level of shared perceptions of supplementary fit was required for beneficial peer-ratings of team effectiveness, as well as for team output (i.e., optimal distinctiveness). We are hopeful that our findings, along with the advanced methodologies used in this study, will stimulate many novel and exciting avenues of research on PC at the team level.

## Ethics Statement

This study was carried out in accordance with the recommendations of ‘The Conjoint Faculties Research Ethics Board (CFREB) of the University of Calgary’ with written informed consent from all subjects. All subjects gave written informed consent in accordance with the Declaration of Helsinki. The protocol was approved by the “The Conjoint Faculties Research Ethics Board (CFREB) of the University of Calgary; REB16-1533.”

## Author Contributions

Conceptualization: KG, YG, GH, and RDC. Data: KG, GH, DO, and HZ. Formal analysis: KG and YG. Investigation: KG, YG, and RDC. Methodology: YG. Project administration: KG, GH, DO, and HZ. Resources: KG, GH, DO, and HZ. Software: YG. Validation: KG, YG, RDC, GH, DO, and HZ. Visualization: KG, YG, RDC, GH, DO, and HZ. Writing original draft: KG, YG, RDC, GH, DO, and HZ. Writing review and editing: KG, YG, RDC, GH, DO, and HZ.

## Conflict of Interest Statement

The authors declare that the research was conducted in the absence of any commercial or financial relationships that could be construed as a potential conflict of interest.

## Footnotes

1 Although not presented here, we would like to note that the results from the partial moderated-mediation model are substantively similar to the results obtained from the full moderated-mediation model (i.e., the same relationships are significant or non-significant when comparing both models).

## References

[B1] Albrecht S. L. Bakker A. B. Gruman J. A. Macey W. H. Saks A. M. (2015). Employee engagement, human resource management practices and competitive advantage: an integrated approach. *J. Organ. Effect.* 2 7–35. 10.1108/JOEPP-08-2014-0042

[B2] Bashshur M. Hernández A. González-Romá V. (2011). When managers and their teams disagree: a longitudinal look at the consequences of differences in perceptions of organizational support. *J. Appl. Psychol.* 96 558–573. 10.1037/a0022675 21341884

[B3] Bell S. T. Villado A. J. Lukasik M. A. Belau L. Briggs A. L. (2011). Getting specific about demographic diversity variable and team performance relationships: a meta-analysis. *J. Manag.* 37 709–743. 10.1177/0149206310365001

[B4] Berry C. M. Carpenter N. C. Barratt C. L. (2012). Do other-reports of counterproductive work behavior provide an incremental contribution over self-reports? A meta-analytic comparison. *J. Appl. Psychol.* 97 613–636. 10.1037/a0026739 22201245

[B5] Blau P. M. (1964). *Exchange and Power in Social Life.* New York, NY: Wiley.

[B6] Bocchino C. C. Hartman B. W. Foley P. F. (2003). The relationship between person-organization congruence, perceived violations of the psychological contract, and occupational stress symptoms. *Consult. Psychol. J. Pract. Res.* 55 203–214. 10.1037/1061-4087.55.4.203

[B7] Bordia P. Restubog S. L. D. Tang R. L. (2008). When employees strike back: investigating mediating mechanisms between psychological contract breach and workplace deviance. *J. Appl. Psychol.* 93 1104–1117. 10.1037/0021-9010.93.5.1104 18808228

[B8] Brewer M. B. (1991). The social self: on being the same and different at the same time. *Pers. Soc. Psychol. Bull.* 17 475–482. 10.1177/0146167291175001

[B9] Byrne D. (1971). *The Attraction Paradigm.* New York, NY: Academic Press.

[B10] Cable D. M. Edwards J. R. (2004). Complementary and supplementary fit: a theoretical and empirical integration. *J. Appl. Psychol.* 89 822–834. 10.1037/0021-9010.89.5.822 15506863

[B11] Chatman J. A. (1989). Improving interactional organizational research: a model of person-organization fit. *Acad. Manag. Rev.* 14 333–349.

[B12] Cole D. A. Maxwell S. E. (2003). Testing mediational models with longitudinal data: questions and tips in the use of structural equation modeling. *J. Abnorm. Psychol.* 112 558–577. 10.1037/0021-843X.112.4.558 14674869

[B13] Dabos G. E. Rousseau D. M. (2013). Psychological contracts and informal networks in organizations: the effects of social status and local ties. *Hum. Resour. Manag.* 52 485–510. 10.1002/hrm.21540

[B14] De Cooman R. Vantilborgh T. Bal M. Lub X. (2016). Creating inclusive teams through perceptions of supplementary and complementary person–team fit: examining the relationship between person–team fit and team effectiveness. *Group Organ. Manag.* 41 310–342. 10.1177/1059601115586910

[B15] De Vos A. Tekleab A. G. (2014). Leaders’ and employees’ psychological contract fulfillment in teams. *Acad. Manag. Proc.* 2014:12928 10.5465/AMBPP.2014.12928abstract

[B16] DeRue D. S. Hollenbeck J. R. (2007). “The search for internal and external fit in teams,” in *Perspectives on Organizational Fit* eds Ostroff C. Judge T. A. (New York, NY: Erlbaum) 259–285.

[B17] DeRue D. S. Morgeson F. P. (2007). Stability and change in person-team and person-role fit over time: the effects of growth satisfaction, performance, and general self-efficacy. *J. Appl. Psychol.* 92 1242–1253. 10.1037/0021-9010.92.5.1242 17845083

[B18] DeShon R. P. Kozlowski S. W. Schmidt A. M. Milner K. R. Wiechmann D. (2004). A multiple-goal, multilevel model of feedback effects on the regulation of individual and team performance. *J. Appl. Psychol.* 89 1035–1056. 10.1037/0021-9010.89.6.1035 15584840

[B19] Edmondson A. (1999). Psychological safety and learning behavior in work teams. *Adm. Sci. Q.* 44 350–383. 10.2307/2666999 22929925

[B20] Edmondson A. C. (2002). *Managing the Risk of Learning: Psychological Safety in Work Teams.* Boston, MA: Harvard Business School.

[B21] Edwards J. R. (1991). “Person-job fit: a conceptual integration, literature review, and methodological critique,” in *International Review of Industrial and Organizational Psychology* eds Cooper C. L. Robertson I. T. (Chichester: Wiley) 283–357.

[B22] Edwards J. R. Cable D. M. Williamson I. O. Lambert L. S. Shipp A. J. (2006). The phenomenology of fit: linking the person and environment to the subjective experience of person-environment fit. *J. Appl. Psychol.* 91 802–827. 10.1037/0021-9010.91.4.802 16834507

[B23] Edwards J. R. Lambert L. S. (2007). Methods for integrating moderation and mediation: a general analytical framework using moderated path analysis. *Psychol. Methods* 12 1–22. 10.1037/1082-989X.12.1.1 17402809

[B24] Edwards J. R. Shipp A. J. (2007). “The relationship between person-environment fit and outcomes: an integrative theoretical framework,” in *Perspectives on Organizational Fit* eds Ostroff C. Judge T. A. (New York, NY: Erlbaum) 209–238.

[B25] Fay D. Shipton H. West M. A. Patterson M. (2015). Teamwork and organizational innovation: the moderating role of the HRM context. *Creat. Innov. Manag.* 24 261–277. 10.1111/caim.12100

[B26] Fisher C. To M. L. (2012). Using experience sampling methodology in organizational behavior. *J. Organ. Behav.* 33 865–877. 10.1002/job.1803

[B27] Geldhof G. J. Preacher K. J. Zyphur M. J. (2014). Reliability estimation in a multilevel confirmatory factor analysis framework. *Psychol. Methods* 19 72–91. 10.1037/a0032138 23646988

[B28] González-Romá V. Fortes-Ferreira L. Peiró J. M. (2009). Team climate, climate strength and team performance. A longitudinal study. *J. Occup. Organ. Psychol.* 82 511–536. 10.1348/096317908X370025

[B29] Gouldner A. W. (1960). The norm of reciprocity: a preliminary statement. *Am. Sociol. Rev.* 25 161–178. 10.1037/a0032138 23646988

[B30] Griep Y. Vantilborgh T. Baillien E. Pepermans R. (2016). The mitigating role of leader-member exchange in reaction to psychological contract violation: a diary study among volunteers. *Eur. J. Work Organ. Psychol.* 25 254–271. 10.1080/1359432X.2015.1046048

[B31] Gruenfeld D. H. Mannix E. A. Williams K. Y. Neale M. A. (1996). Group composition and decision making: how member familiarity and information distribution affect process and performance. *Organ. Behav. Hum. Decis. Process.* 67 1–15. 10.1006/obhd.1996.0061

[B32] Guan Y. Deng H. Risavy S. D. Bond M. H. Li F. (2011). Supplementary fit, complementary fit, and work-related outcomes: the role of self-construal. *Appl. Psychol. Int. Rev.* 60 286–310. 10.1111/j.1464-0597.2010.00436.x

[B33] Hackman J. R. (1987). “The design of work teams,” in *Handbook of Organizational Behavior* ed. Lorsch J. (New York, NY: Prentice Hall) 315–342.

[B34] Hansen S. D. Griep Y. (2016). “Psychological contracts,” in *Handbook of Employee Commitment* ed. Meyer J. (Northampton, MA: Edward Elgar Publishing) 119–134.

[B35] Ho V. T. Levesque L. L. (2005). With a little help from my friends (and substitutes): social referents and influence in psychological contract fulfillment. *Organ. Sci.* 16 275–289. 10.1287/orsc.1050.0121

[B36] Ho V. T. Rousseau D. M. Levesque L. L. (2006). Social networks and the psychological contract: structural holes, cohesive ties, and beliefs regarding employer obligations. *Hum. Relat.* 59 459–481. 10.1177/0018726706065370

[B37] Hoffman L. R. Maier N. R. (1959). The use of group decision to resolve a problem of fairness. *Pers. Psychol.* 12 545–559. 10.1111/j.1744-6570.1959.tb01342.x

[B38] Hofmann D. A. Griffin M. A. Gavin M. B. (2000). “The application of hierarchical linear modeling to organizational research,” in *Multilevel Theory, Research, and Methods in Organizations: Foundations, Extensions, and New Directions* eds Klein K. J. Kozlowski S. W. J. (San Francisco, CA: Jossey-Bass) 467–511.

[B39] James L. A. James L. R. (1989). Integrating work environment perceptions: explorations into the measurement of meaning. *J. Appl. Psychol.* 74 739–751. 10.1037/0021-9010.74.5.739

[B40] James L. R. Demaree R. G. Wolf G. (1984). Estimating within-group interrater reliability with and without response bias. *J. Appl. Psychol.* 69 85–98. 10.1037/0021-9010.69.1.85

[B41] Klein C. DiazGranados D. Salas E. Le H. Burke C. S. Lyons R. (2009). Does team building work? *Small Group Res.* 40 181–222. 10.1177/1046496408328821

[B42] Klein K. J. Kozlowski S. W. J. (2000). “A multilevel approach to theory and research in organizations contextual, temporal, and emergent processes,” in *Multilevel Theory, Research, and Methods in Organizations: Foundations, Extensions, and New Directions* eds Kozlowski S. W. J. Klein K. J. (San Francisco, CA: Jossey-Bass) 3–90.

[B43] Kozlowski S. W. J. Chao G. T. Grand J. A. Braun M. T. Kuljanin G. (2013). Advancing multilevel research design: capturing the dynamics of emergence. *Organ. Res. Methods* 16 581–615. 10.1177/1094428113493119

[B44] Kristof A. L. (1996). Person-organization fit: an integrative review of its conceptualizations, measurement, and implications. *Pers. Psychol.* 49 1–49. 10.1111/j.1744-6570.1996.tb01790.x

[B45] Kristof-Brown A. Barrick M. R. Stevens C. K. (2005). When opposites attract: a multi-sample demonstration of complementary person-team fit on extraversion. *J. Pers.* 73 935–958. 10.1111/j.1467-6494.2005.00334.x 15958140

[B46] Kristof-Brown A. L. Guay R. P. (2011). “Person-environment fit,” in *APA Handbook of Industrial and Organizational Psychology* ed. Zedeck S. (Washington, DC: American Psychological Association) 3–50.

[B47] Kristof-Brown A. L. Zimmerman R. D. Johnson E. C. (2005). Consequences of individuals’ fit at work: a meta-analysis of person-job, person-organization, person-group, and person-supervisor fit. *Pers. Psychol.* 58 281–342. 10.1111/j.1744-6570.2005.00672.x

[B48] Kristof-Brown A. L. Seong J. Y. Degeest D. S. Park W. W. Hong D. S. (2014). Collective fit perceptions: a multilevel investigation of person–group fit with individual-level and team-level outcomes. *J. Organ. Behav.* 35 969–989. 10.1002/job.1942

[B49] Laulié L. Tekleab A. G. (2016). A multi-level theory of psychological contract fulfillment in teams. *Group Organ. Manag.* 41 658–698. 10.1177/1059601116668972

[B50] LeBreton J. M. Senter J. L. (2008). Answers to 20 questions about interrater reliability and interrater agreement. *Organ. Res. Methods* 11 815–852. 10.1177/1094428106296642

[B51] Loughry M. L. Ohland M. W. Moore D. D. (2007). Development of a theory-based assessment of team member effectiveness. *Educ. Psychol. Meas.* 67 505–524. 10.1177/0013164406292085

[B52] MacKinnon D. P. (2008). *Introduction to Statistical Mediation Analysis.* New York, NY: Lawrence Erlbaum Associates.

[B53] Marks A. (2001). Developing a multiple foci conceptualization of the psychological contract. *Employee Relat.* 23 454–469. 10.1108/EUM0000000005897

[B54] Mohammed S. Ferzandi L. Hamiilton K. (2010). Metaphor no more: a 15-year review of the team mental model construct. *J. Manag.* 36 876–910. 10.1177/0149206309356804

[B55] Morgeson F. P. Hofmann D. A. (1999). The structure and function of collective constructs: implications for multilevel research and theory development. *Acad. Manag. Rev.* 24 249–265. 10.5465/AMR.1999.1893935

[B56] Morrison E. W. Robinson S. L. (1997). When employees feel betrayed: a model of how psychological contract violation develops. *Acad. Manag. Rev.* 22 226–256. 10.5465/AMR.1997.9707180265

[B57] Muchinsky P. M. Monahan C. J. (1987). What is person-environment congruence? Supplementary versus complementary models of fit. *J. Vocat. Behav.* 31 268–277. 10.1016/0001-8791(87)90043-1

[B58] Muthén L. K. Muthén B. O. (2013). *Mplus User’s Guide* 7th Edn. Los Angeles, CA: Muthén & Muthén.

[B59] Nemeth C. J. (1986). Differential contributions of majority and minority influence. *Psychol. Rev.* 93 23–32. 10.1037/0033-295X.93.1.23

[B60] O’Leary-Kelly A. M. Henderson K. E. Anand V. Ashforth B. E. (2014). Psychological contracts in a nontraditional industry: exploring the implications for psychological contract development. *Group Organ. Manag.* 39 326–360. 10.1177/1059601114525851

[B61] O’Neill T. Hoffart G. McLarnon M. Woodley H. Eggermont M. Rosehart W. (2017). Constructive controversy and reflexivity training promotes effective conflict profiles and outcomes in student learning teams. *Acad. Manag. Learn. Educ.* 16 257–276. 10.5465/amle.2015.0183

[B62] O’Neill T. A. Allen N. J. Hastings S. E. (2013). Examining the “pros” and “cons” of team conflict: a team-level meta-analysis of task, relationship, and process conflict. *Hum. Perform.* 26 236–260. 10.1080/08959285.2013.795573

[B63] Ostroff C. (2012). “Person-environment fit in organizational settings,” in *The Oxford Handbook of Organizational Psychology* ed. Kozlowski S. W. J. (New York, NY: Oxford University Press) 373–408.

[B64] Piasentin K. A. Chapman D. S. (2007). Perceived similarity and complementarity as predictors of subjective person-organization fit. *J. Occup. Organ. Psychol.* 80 341–354. 10.1348/096317906X115453

[B65] Podsakoff P. M. MacKenzie S. B. Podsakoff N. P. (2012). Sources of method bias in social science research and recommendations on how to control it. *Annu. Rev. Psychol.* 63 539–569. 10.1146/annurev-psych-120710-100452 21838546

[B66] Preacher K. J. Rucker D. D. Hayes A. F. (2007). Addressing moderated mediation hypotheses: theory, methods, and prescriptions. *Multivar. Behav. Res.* 42 185–227. 10.1080/00273170701341316 26821081

[B67] Rousseau D. (1995). *Psychological Contracts in Organizations: Understanding Written and Unwritten Agreements.* Thousand Oaks, CA: Sage Publications.

[B68] Rousseau D. M. (1989). Psychological and implied contracts in organizations. *Employee Respons. Rights J.* 2 121–139. 10.1007/BF01384942

[B69] Rousseau D. M. (2001). Schema, promise and mutuality: the building blocks of the psychological contract. *J. Occup. Organ. Psychol.* 74 511–541. 10.1348/096317901167505

[B70] Salas E. Goodwin G. F. Burke C. S. (2008). *Team Effectiveness in Complex Organizations: Cross-Disciplinary Perspectives and Approaches.* New York, NY: Routledge.

[B71] Salas E. Shuffler M. L. Thayer A. L. Bedwell W. L. Lazzara E. H. (2015). Understanding and improving teamwork in organizations: a scientifically based practical guide. *Hum. Resour. Manag.* 54 599–622. 10.1002/hrm.21628

[B72] Seong J. Kristof-Brown A. L. (2012). Testing multidimensional models of person-group fit. *J. Manag. Psychol.* 27 536–556. 10.1108/02683941211252419

[B73] Seong J. Y. Choi J. N. (2014). Effects of group-level fit on group conflict and performance: the initiating role of leader positive affect. *Group Organ. Manag.* 39 190–212. 10.1177/1059601113517138

[B74] Seong J. Y. Kristof-Brown A. L. Park W. W. Hong D. S. Shin Y. (2015). Person-group fit: diversity antecedents, proximal outcomes, and performance at the group level. *J. Manag.* 41 1184–1213. 10.1177/0149206312453738

[B75] Shipp A. J. Jansen K. J. (2011). Reinterpreting time in fit theory: crafting and recrafting narratives of fit in medias res. *Acad. Manag. Rev.* 36 76–101. 10.5465/amr.2009.0077

[B76] Siemsen E. Roth A. Oliveira P. (2010). Common method bias in regression models with linear, quadratic, and interaction effects. *Organ. Res. Methods* 13 456–476. 10.1177/1094428109351241

[B77] Stasser G. Stewart D. D. Wittenbaum G. M. (1995). Expert roles and information exchange during discussion: the importance of knowing who knows what. *J. Exp. Soc. Psychol.* 31 244–265. 10.1006/jesp.1995.1012

[B78] Vantilborgh T. Bidee J. Pepermans R. Griep Y. Hofmans J. (2016). Antecedents of psychological contract breach: the role of job demands, job resources, and affect. *PLOS ONE* 11:e0154696. 10.1371/journal.pone.0154696 27171275 PMC4865204

[B79] Zhao H. Wayne S. J. Glibkowski B. C. Bravo J. (2007). Impact of psychological contract breach on work-related outcomes: a meta-analysis. *Pers. Psychol.* 60 647–680. 10.1111/j.1744-6570.2007.00087.x

